# Traumatic fracture of central venous catheter resulting in potential migration of distal fragment: a case report

**DOI:** 10.1186/1757-1626-1-394

**Published:** 2008-12-14

**Authors:** Shailendra Deep, Sanjay Deshpande, Philip Howe

**Affiliations:** 1 Dept of Anaesthetic, Ysbyty Gwynedd, Bangor, LL57 2PW, UK; 2 Dept of Anaesthetic, South Tyneside District Hospital, South Shields, NE34 1LP, UK; 3 Dept of Intensive Care, South Tyneside District Hospital, South Shields, NE34 1LP, UK

## Abstract

We report a surgical retrieval of an indwelling portion of a traumatic rupture of the Central venous catheter following hair cutting by a confused patient secondary to Postoperative cognitive dysfunction. He had a dynamic compression screw for fixation of fractured neck of femur after previously failed surgical procedure. The second procedure was complicated with major blood loss, which required central venous and arterial line insertion for intra-operative and post-operative management. The patient was discharged to the ward following an uneventful stay on intensive care. While on the ward, he decided to trim his hair and in the process he inadvertently cut through the right internal jugular catheter. Complications and management resulting from embolisation of central line are reviewed.

## Introduction

Percutaneous insertion of central venous catheters is a routine hospital practice. Complications of the procedure occur either during insertion (arterial puncture resulting in bleeding, pneumothorax, cardiovascular side-effects) and/or during maintenance of the line such as infection, thrombosis or other mechanical risks[1] Mechanical complications such as catheter fractures and catheter migration [2-5] is rare with an estimated rate of 0.1%[6]. Accidental embolisation of a fragment of central venous catheter is a rare but potentially serious complication. We describe an unusual traumatic fracture of internal jugular line during hair cutting resulting in subsequent migration of the indwelling catheter, which was retrieved surgically without incident.

## Case report

An 80-year-old male patient with significant morbidity, which included Hypertension, Ischemic Heart Disease, Atrial Fibrillation, Chronic Obstructive Pulmonary Disease, Cirrhosis of the Liver and Hepatitis C was admitted to the Orthopaedic ward with a fracture of the left hip following an alleged fall. He had no other injuries.

Following thorough investigations and optimisation, a left Dynamic Hip Screw procedure was planned and carried out under spinal anaesthesia. The surgical procedure was uneventful, but postoperatively, he developed a wound infection, which resulted in an unstable hip joint. The surgeons proceeded to re-operate and a DCS (Dynamic Compression Screw) procedure was performed to stabilise the hip. The surgery was complicated by massive blood loss estimated at 3,500 ml. He required 18 units of Packed Red Cells, 4 units of FFP and 1 unit of Platelets for haemodynamic stabilisation.

After successful haemostasis and recovery from anaesthesia, the patient was transferred to the High Dependency Unit for monitoring and further management. A right Internal Jugular central line and a radial arterial line were in situ.

The patient spent three days in the High dependency unit where apart from being mentally confused, he remained haemodynamically stable. He was transferred back to the ward on the third postoperative day. On arrival in the ward he remained confused possibly due to Postoperative Cognitive Dysfunction.

Whilst in the ward, he decided to trim his hair and in the process he inadvertently cut through the right internal jugular catheter. The Nurses during routine observations found the cut portion of the central line on his bedside locker.

**Figure 1:**
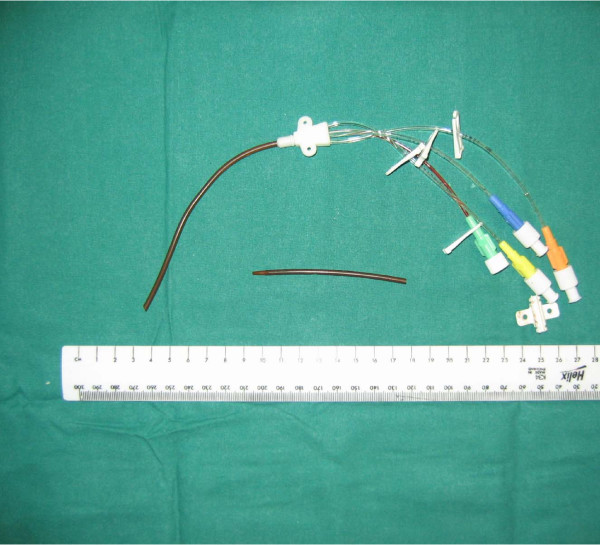
(Central Venous Catheter).

**Figure 2:**
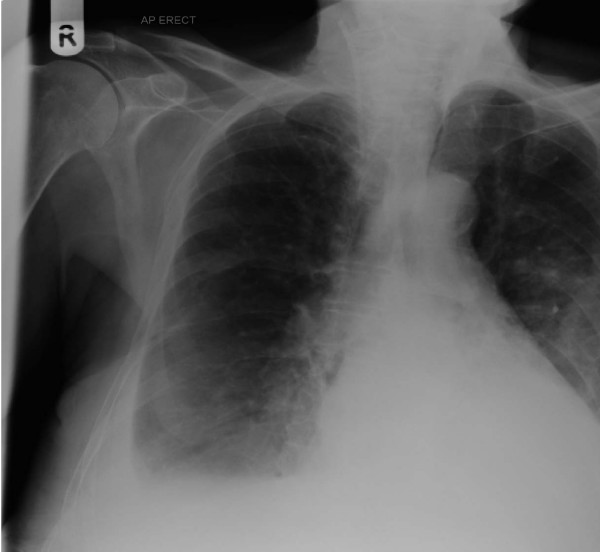
(An X-Ray).

On examination of the cut end of the catheter, there was a clean 45 degree cut at its distal end (figure 1). He remained asymptomatic; on clinical examination the tip of catheter was palpable deep to the skin of the neck. An X-ray of the chest (figure 2) confirmed 8 cm of the line within the internal jugular vein. Surgical exploration to remove the catheter remnant was uneventful.

## Discussion

Embolisation of a polyethylene catheter inserted from cubital vein to right atrium was first reported in 1954. [7]

In a series of 220 documented cases of catheter embolism, 71% had morbidity in spite of catheter retrieval, and 38% had mortality, as catheter fragment was not removed. Causes of death incriminated were pericardial tamponade, myocardial perforation, sepsis, endocarditis, thrombosis, pulmonary embolism, myocardial infarction and arrhythmias. [8]

Shearing or fracture of central venous catheter is a recognised complication. One centre reports an incidence of 2.5% over a five year period; however, these incidents were all in neonates [9]. Causes that have been described are shearing by the introducer needle during insertion [9], high pressure within the catheter due to bolus infusion [9] fracturing of external portion by patient's body movement (Mostly infants) [10], during removal of a stuck catheter mostly by fibrin sheath formation around the catheter [11], and weakening of the catheter tip by movement of tricuspid valve and right ventricular motion [10], or mechanical forces between the clavicle and the first rib [12]. In this presented case the patient cut through the internal jugular catheter with scissors while trimming his hair.

Catheter fatigue from prolonged use contributes to in-situ fracture; fragmentation and distal embolisation [13], the catheter fragment migrate distally along the central vein and finally lodging in the vena cava, right atrium, right ventricle, pulmonary artery and its branches. The final site of lodgement depends on their length, weight, and the material stiffness. Catheter tip migration can occur especially if the patient vomits or if he coughs vigorously or sneezes excessively. However, migration of these broken fragments are associated with very serious complication like embolisation into pulmonary artery or myocardial perforation or necrosis culminating in temponade, myocardial infarction, valvular perforation, arrhythmia, cardiac arrest and death. The foreign body can act as a nidus for thrombus formation with resultant pulmonary embolism. Infectious complications include endocarditis, secondary infection of thrombus, mycotic aneurysm, and pulmonary abscesses. Interventional radiological technique (using loop snares, hooked guide wire and Fogarty balloon catheters) are used to retrieve the catheter and in rare cases surgical intervention may be needed. In our patient the catheter was retrieved surgically as catheter was lying subcutaneously, not completely embolised.

## Consent

We are unable to obtain consent from the patient, as he is deceased and we are unable to contact his relatives. The hospital legal advisor has approved the x-ray report to be published for educational purposes.

## Competing interests

The authors declare that they have no competing interests.

## Authors' contributions

SD (Shailendra Deep) wrote this case representation and search the references. SD (Sanjay Deshpande) guided and gave the finer correction of manuscript of case representation. PH (Phil Howe) helped in writing manuscript and provided pictures in appropriate format.
